# IsoTools: a flexible workflow for long-read transcriptome sequencing analysis

**DOI:** 10.1093/bioinformatics/btad364

**Published:** 2023-06-02

**Authors:** Matthias Lienhard, Twan van den Beucken, Bernd Timmermann, Myriam Hochradel, Stefan Börno, Florian Caiment, Martin Vingron, Ralf Herwig

**Affiliations:** Department of Computational Biology, Max Planck Institute for Molecular Genetics, 14195 Berlin, Germany; Department of Toxicogenomics, Maastricht University, Maastricht 6229ER, The Netherlands; Sequencing Core Unit, Max Planck Institute for Molecular Genetics, 14195 Berlin, Germany; Sequencing Core Unit, Max Planck Institute for Molecular Genetics, 14195 Berlin, Germany; Sequencing Core Unit, Max Planck Institute for Molecular Genetics, 14195 Berlin, Germany; Department of Toxicogenomics, Maastricht University, Maastricht 6229ER, The Netherlands; Department of Computational Biology, Max Planck Institute for Molecular Genetics, 14195 Berlin, Germany; Department of Computational Biology, Max Planck Institute for Molecular Genetics, 14195 Berlin, Germany

## Abstract

**Motivation:**

Long-read transcriptome sequencing (LRTS) has the potential to enhance our understanding of alternative splicing and the complexity of this process requires the use of versatile computational tools, with the ability to accommodate various stages of the workflow with maximum flexibility.

**Results:**

We introduce IsoTools, a Python-based LRTS analysis framework that offers a wide range of functionality for transcriptome reconstruction and quantification of transcripts. Furthermore, we integrate a graph-based method for identifying alternative splicing events and a statistical approach based on the beta-binomial distribution for detecting differential events. To demonstrate the effectiveness of our methods, we applied IsoTools to PacBio LRTS data of human hepatocytes treated with the histone deacetylase inhibitor valproic acid. Our results indicate that LRTS can provide valuable insights into alternative splicing, particularly in terms of complex and differential splicing patterns, in comparison to short-read RNA-seq.

**Availability and implementation:**

IsoTools is available on GitHub and PyPI, and its documentation, including tutorials, CLI, and API references, can be found at https://isotools.readthedocs.io/.

## Graphical abstract

**Graphical Abstract:**
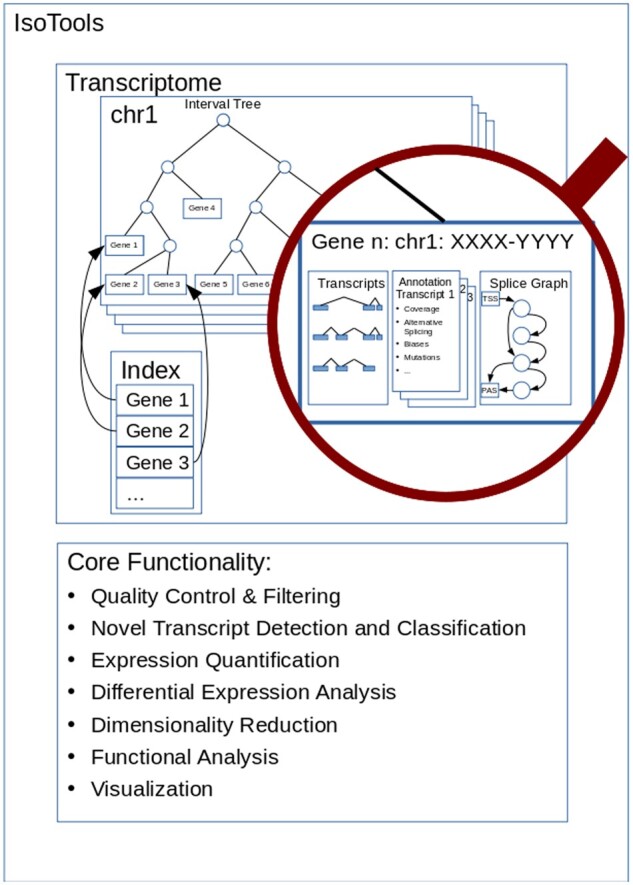

## 1 Introduction

Long-read transcriptome sequencing (LRTS) allows for full-length sequencing of expressed transcripts. In contrast to short-read RNA-seq, this technology does not require fragmentation of transcripts and thereby avoids introduction of errors due to alignment ambiguity and other technical artifacts. This is particularly relevant for the analysis of complex alternative splicing, since short reads cannot be assigned reliably to transcripts over longer genomic distances, and thus facilitates the identification of novel transcripts (Kanitz *et al.* 2015, Zhang *et al.* 2017, Byrne *et al.* 2019, Sarantopoulou *et al.* 2021).

With the LRTS Iso-Seq protocol from PacBio, besides direct cDNA/RNA sequencing by Oxford Nanopore, a technology has emerged that holds the promise to improve transcript identification and quantification. While hybrid sequencing approaches have been suggested in the past to combine detection (LRTS) and quantification (RNA-seq) of transcripts (Au *et al.* 2013), recent advances in accuracy and throughput facilitate direct quantification of transcripts and splicing events from LRTS data alone (Pardo-Palacios *et al.* 2021).

LRTS offers a wide range of applications for both model and nonmodel organisms. It has been used to identify important gene structures and coding regions for poorly annotated nonmodel organisms (Abdel-Ghany *et al.* 2016, Clavijo *et al.* 2017). For model organisms such as humans, LRTS is commonly used to explore gene models, recover novel transcripts, and alternative splicing events (ASEs), and to quantify transcript expression, with or without the integration of short-read data (Au *et al.* 2013, Sahlin *et al.* 2018). Very recently, LRTS has been combined with single-cell technology, to explore and characterize splicing on the level of cell types (Mincarelli *et al.* 2020, Zheng *et al.* 2020, Joglekar *et al.* 2021).

Previous studies have established a moderate correlation between LRTS and short-read measurements at the gene level, however, this correlation diminishes significantly when evaluating transcript expression (Reese and Mortazavi 2020, Wyman *et al.* 2020, Leung *et al.* 2021).

To meet the demands of high-throughput transcriptome sequencing, various tools and pipelines have been developed to analyze and interpret the data. Tools such as TALON, FLAIR, IsoQuant, and Bambu focus on transcriptome reconstruction and quantification from LRTS datasets (Tang *et al.* 2020, Wyman *et al.* 2020, Chen *et al.* 2021, Prjibelski *et al.* 2023). SQANTI3 focuses on characterization of known and novel transcripts obtained from these tools, and thus facilitates evaluation. The Swan library (Reese and Mortazavi 2020) implements visualization functionality, and a statistical test based on a negative binomial model for differential expression analysis on transcript level using LRTS.

Here, we present IsoTools, a novel tool for LRTS analysis that integrates additional features and provides unprecedented flexibility of the different analysis modules in order to facilitate highly explorative and integrative analysis of the complex sequencing data. IsoTools is a modular Python framework that integrates all relevant information from identified transcripts and reference annotation, and provides a variety of novel analysis functions to explore, analyze, and interpret the data. For example, for differential splicing, the software offers on the one hand the transcript-level quantification of isoforms and on the other hand several statistical tests are provided that allow splice event-level quantification that can be directly compared with short-read data. The software emphasizes modularity, allowing users to customize their analysis workflow and integrate with external tools. For example, IsoTools enables the import of transcripts detected by other tools and their further analysis, visualization, or filtering. We designed IsoTools to be user-friendly with a clear and intuitive interface, detailed documentation, and tutorials to make it accessible for users with varying computational expertise. We describe the major components of IsoTools in transcriptome reconstruction, quantification, refined classification of novel transcripts, and differential splicing. Additionally, we performed a methods comparison for transcriptome reconstruction with four other tools and showed that the detection of isoforms is largely dependent on the different filtering settings with IsoTools adapting to different scenarios. To demonstrate the tools capability in the context of drug response, we generated LRTS data from human hepatocytes treated with the histone deacetylase (HDAC) inhibitor valproic acid (VPA). We compared the quantification of LRTS with RNA-seq on the same samples both on the recovered transcript level as well as on the splice event level. Our analysis revealed known and novel splicing events with *in vivo* relevance as confirmed by healthy human liver tissue. Overall, IsoTools is a versatile and powerful tool for LRTS analysis with a workflow of unprecedented flexibility and modularity that can easily be adapted to the specific needs of the user.

## 2 Materials and methods

### 2.1 Sample preparation and IsoSeq sequencing

The preparation of the samples processed here is described in Wolters *et al.* (2018). In brief, human hepatocytes were exposed to 15 mM VPA or 1% EtOH (CTL) and sampled after one, two, and three days of treatment. For the fourth time point, VPA was washed out after day 3, and cells were resampled after three additional days. We prepared cDNA samples in triplicate for each time point and condition. Using the PacBio Sequel II platform, we pooled and sequenced the VPA treated and control samples, using one 8M SMRT cell per pool. After confirming sample integrity and cDNA generation, we followed the PacBio IsoSeq library preparation protocol (Supplementary Method S1) and sequenced the samples on two Sequel II SMRT cells, resulting in 6.7 and 3.9 M polymerase reads for CTL- and VPA-treated samples, respectively.

IsoSeq subreads were processed using Iso-Seq v3.4 pipeline with recommended parameters (Supplementary Method S2), resulting in 2.6 and 4.3 M HiFi poly-A reads for VPA and CTL samples, respectively, with an average length of 3.6 and 3.9 K bases. 96.6% and 95.5% of these HiFi reads have an error rate of less than 1%. The flnc reads were aligned to the human genome GRCh38.p13 using minimap2 and further analysis was performed using IsoTools, taking about 67 min on a single CPU core, using 20 GB RAM. The LRTS data are available from ENA under accession number PRJEB46194.

### 2.2 Complementary data

#### 2.2.1 RNA-seq

RNA-seq data for the primary hepatocytes and liver samples were downloaded from ENA accession numbers PRJEB22198 and PRJEB35350, respectively. The short reads were aligned to the human reference genome GRCH38.p13 using STAR aligner version 2.7.6a (Dobin *et al.* 2013), with provided gff annotation from GENCODE release 36 including annotation of nonchromosomal scaffolds (Frankish *et al.* 2019). For hepatocytes, we merged all alignments from the same time point, to resemble the IsoSeq samples. We used rsem v1.3.1 (Li and Dewey 2011) with the transcriptome alignment from STAR to obtain read counts on gene and transcript level. Next, we used rMATS rmats-turbo v4.1.1 (Shen *et al.* 2014) to find differentially spliced events between VPA-treated hepatocytes and control, either using events calculated by rMATS, or by providing events generated by IsoTools from the GENCODE reference as well as from IsoSeq LRTS.

#### 2.2.2 ENCODE CAGE data

We downloaded CAGE TSS peaks for HepG2 cells from the ENCODE data portal (Davis *et al.* 2018) (https://www.encodeproject.org/) with the following identifiers: ENCFF089AFK, ENCFF220OWX, ENCFF241CGD, ENCFF248QKX, ENCFF373BNI, ENCFF419FNU, ENCFF875ILB, and ENCFF885VJU.

### 2.3 Computational approaches

#### 2.3.1 Saturation analysis

We implemented a novel model-based approach to assist the user with estimating the required depth of sequencing. The model is based on the cumulative distribution function of a negative binomial distribution, with three parameters *n*, *r*, and *p*: The sampling probability *p* depends on the cellular concentration of the transcript in the samples. A highly expressed transcript is more likely to be covered compared with a transcript with few RNA molecules per cell. The model assumes the sampling probability to be proportional to the RNA concentration. The second parameter, *r*, corresponds to the minimum required coverage to confidently call a transcript impacts the chance of a transcript to be discovered. The PacBio IsoSeq clustering pipeline requires two copies of a transcript to report it. However, depending on the application, it may be appropriate to reduce or increase this threshold (see Supplementary Method S3 and Supplementary Fig. S2). The last parameter, *n*, corresponds to the sequencing depth. If two parameters are given, the model can be used to determine the missing parameter. For example, if the concentration of a transcript of interest can be estimated, and the required minimum coverage is chosen, the model can suggest the required sequencing depth.

#### 2.3.2 Transcriptome reconstruction

To reconstruct the transcriptome from aligned reads, IsoTools groups reads with the same intron chain into transcripts and groups transcripts sharing at least one splice junction into genes. Similarly, genes sharing splice junctions with a reference gene are assigned to that reference gene. Unspliced transcripts are assigned to a gene if they overlap at least 50%. Next, IsoTools corrects ambiguous splice sites by comparing their positions to the reference annotation. Specifically, if both the donor and acceptor sites of a splice junction are shifted in the same direction by the same number of bases compared with the reference, IsoTools uses the reference positions. To determine the positions of transcription start sites (TSSs) and polyadenylation sites (PASs), IsoTools employs a gene-wise peak calling approach to identify the most prominent start and end positions of reads. For each transcript, we assign the TSS and PAS by identifying the peak closest to the most read start and end positions, respectively. By using this method, IsoTools standardizes the positions of TSSs and PASs across all transcripts of a given gene, without being biased by the reference annotation. Finally, IsoTools computes quality control (QC) values for each transcript based on various features, to facilitate the detection and subsequent filtering of technical artifacts (see Supplementary Method S4).

#### 2.3.3 Filtering query syntax

Transcript filtering is implemented as a query syntax, based on logical combinations of named “tags,” by convention, a single word in capital letters. These tags are defined Python expressions, which are evaluated in the context of the transcript dictionary, so it may depend on all metrics and properties of the transcript. IsoTools provides predefined tags, covering technical artifacts, but also the novelty categories, and properties of the reference annotation. In Supplementary Method S4, we describe how these default definitions have been derived. Users may adapt the expressions, or add custom tags with custom expressions, to further extend the filtering framework. These tags can then be logically combined in a query string, which is passed to the analysis or export functions in IsoTools, and evaluated for each transcript. In addition, the user may specify a genomic region, and a minimum or maximum read coverage.

#### 2.3.4 Definition and classification of binary ASEs

In analogy to the commonly used definition on splice graphs (Sammeth 2009), we define binary ASEs based on bubbles in the segment graph of a gene. In a segment graph, nodes represent disjoint exonic segments and edges imply that the two exonic segments succeed one another in one or more transcripts. The segment graph is bidirected, as each node has a set of incoming and outgoing edges, and ordered by the genomic position, meaning an edge from node $x_{i}$ to node $x_{j}$ is only allowed if $x_{i}<x_{j}$, e.g. the genomic end position of $x_{i}$ is smaller or equal to the start position of $x_{j}$. If two succeeding segments are separated by an intron, the edge represents a splice junction. On the other hand, if the genomic end position of the preceding segment corresponds to the genomic start position of the succeeding segment, the edge is called internal. This implies either an alternative preceding or succeeding segment, connected by a splice junction edge.

Bubbles are structures in the segment graph, with two paths starting in a common segment $x_{s}$ and ending in a common segment $x_{e}$, but the paths do not share any segments except $x_{s}$ and $x_{e}$ (cf. Fig. 3A–C). We define the “primary path” as the path for which the outgoing edge from $x_{s}$ exceeds the outgoing edge of the other path, which in turn is called the “alternative path.” We further categorize the alternative path in five different classes: the alternative is classified as mutually exclusive (ME) if the primary path from $x_{s}$ to $x_{e}$ contains at least one additional segment $x_{ME}$. The definition of the other classes depends on whether the outgoing edge of $x_{s}$ and the incoming edge of $x_{e}$ on the alternative path correspond to splice junctions or internal edges (within an exon). Alternative paths are called exon skipping (ES) if both edges are splice junctions, and as intron retention (IR) if both edges are internal. If one of the edges is a splice junction and the other internal, the alternative is classified as 5' or 3' alternative splice site (5AS and 3AS). For each primary path, we group all alternative paths of the same category, and find the set of transcripts *A* supporting one of the alternatives and *B* supporting the primary. This definition results in a finite set of classified binary ASEs for each gene. They can be quantified by the percent spliced-in index (PSI), defined as the number of long reads supporting transcripts from *A* over the total number of reads, supporting transcripts from *A* or *B*.

#### 2.3.5 Statistical tests for differential splicing

We implemented three statistical tests for differential splicing. The first two, likelihood ratio test with binomial model and two-proportions *z*-test, apply if two individual samples are compared.

For the “binomial likelihood ratio test,” the number of supporting reads is modeled with a binomial distribution. The likelihood ratio test is specified by the statistic where $\ell_{1}=ln(B(k_{1}|{\hat{p}}_{1},n_{1}))+ln(B(k_{2}|{\hat{p}}_{2},n_{2}))$ and are the maximized log-likelihoods under the alternative $H_{1}$ and the null hypothesis $H_{0}$. B(n∣p,n) is the probability mass function of the binomial distribution, which is maximal at the empirical PSI values ${\hat{p}}_{i}=\frac{k_{i}}{n_{i}}$ for sample *i*, and $\hat{p}=\frac{k_{1}+k_{2}}{n_{1}+n_{2}}$ for both samples combined.


(1) $$\Lambda =-2(\ell_{1}-\ell_{0})∼\chi^{2},$$

$$\ell_{0}=ln(B(k_{1}+k_{2}|\hat{p},n_{1}+n_{2}))$$

Alternatively, when the sample size is large enough (n>30 reads), the “two-proportions *z*-test” can be used as an approximation. The test is specified by the statistic where $n_{i}=k_{i}+l_{i}$ is the total number of reads of sample i∈[1,2] covering the event. $k_{i}$ and $l_{i}$ are the number of reads supporting the alternative (set *A*) and primary (set *B*) variants, respectively. Both tests yield very similar results (*P*-values *r* = .999, see Supplementary Fig. S13).


(2) $$z=\frac{\hat{p_{1}}-\hat{p_{2}}}{\sqrt{\hat{p}(1-\hat{p})(\frac{1}{n_{1}}+\frac{1}{n_{2}})}}∼N(0,1),$$

In the context of differential splicing analysis between two groups of samples, the PSI values may vary within each group, leading to overdispersion and rendering the binomial distribution unsuitable. To address this, IsoTools implements a “beta-binomial mixture likelihood ratio test,” which captures the variability within the two groups by modeling the number of supporting reads with a beta-binomial mixture distribution. The probability parameter *p* in the binomial distribution is modeled with a beta distribution Beta(a,b), flexibly representing variations in the PSI values between the samples. In IsoTools, the maximum log-likelihood parameters $\hat{a}$ and $\hat{b}$ are determined numerically by a quasi-Newton optimization method (LM-BFGS from SciPy [Virtanen *et al.* 2020]). The beta-binomial model has been recently applied to model alternative splicing in RNA-seq data, e.g. in the context of identifying rare splicing events in tissue data (Mertes *et al.* 2021) and to identify subtypes of cancer (Wang *et al.* 2023).

In addition to ASEs, all tests defined above can also be applied to detect differential usage of transcription start and poly-A sites. In this case, all transcripts supporting a particular start/poly-A site are considered the alternative set *B*, whereas all other transcripts constitute the primary set *A*. With all tests, multiple testing correction is applied (Benjamini and Hochberg, 1995).

## 3 Results

### 3.1 IsoTools streamlines transcriptome data for efficient exploration and filtering

IsoTools handles the transcriptome reconstruction process by importing aligned full-length reads, correcting for splice junction ambiguities, grouping the reads into transcripts, and grouping transcripts into genes. Each gene’s splicing structure is then represented as a segment graph, with nodes representing exonic segments and edges indicating the sequence of exons within a gene’s transcripts (Section 2).

In addition to the splicing structure, IsoTools extracts a range of information from the alignments, including the number of reads per transcript, several QC metrics, and mutation information, and integrates this information with data from the reference genome and annotation. The resulting tree-based data structure (Supplementary Fig. S1) can be accessed by genomic position or gene identifier and facilitates exploring the data at multiple levels of detail, from individual nucleotides to transcriptome-wide statistics. The transcriptome can also be easily exported or imported from or to gtf format for compatibility with other tools.

**Figure 1.**
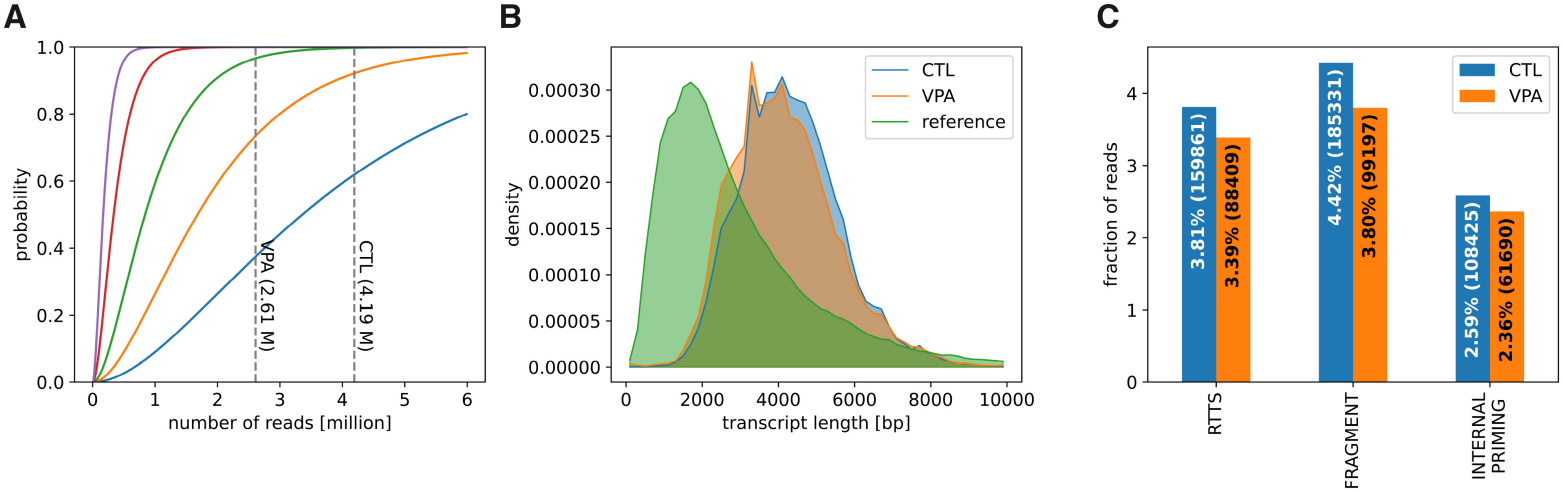
(A) Probability of observing a transcript depending on the cellular concentration of 0.5 (blue line), 1 (orange), 2 (green), 5 (red), and 10 TPM (purple) and sequencing depth at detection threshold of two reads. Dashed horizontal lines represent the sequencing depth of the VPA and CTL IsoSeq samples. (B) Read length distribution compared with level 1 GENCODE transcripts. (C) Fraction of reads affected by different technical artifacts.

To validate the method, we generated datasets from human hepatocytes treated with the HDAC inhibitor VPA and untreated cells (CTL), yielding 2 615 181 and 4 200 885 aligned full-length non-chimeric poly-A HiFi reads, respectively. Our saturation model (Section 2) indicated that for both samples, the probability of observing even rare transcripts (one TPM) was over 70%, and for transcripts expressed at two TPM or more, the probability of observing at least two reads was close to 100%. These results demonstrate the saturation of transcript discovery at the given concentration and detection threshold (Fig. 1A).

To minimize the impact of technical artifacts and improve the accuracy of transcript identification, IsoTools employs various QC measures, such as the transcript length distribution, to identify systematic depletion of shorter or longer transcripts in comparison to the reference (Fig. 1B). Additionally, QC methods comprise detection of technical artifacts, such as internal priming, reverse transcriptase template switching (RTTS), and truncation. Based on QC metrics, we implemented specific filter expressions for these artifacts, to tag affected transcripts (Section 2). For subsequent analyses, visualizations, and data exports, the user can remove affected transcripts according to these tags. In order to find reasonable thresholds for the filter expressions, we made use of additional information such as CAGE TSS peaks, and compared QC metrics on the most credible GENCODE transcripts with support level 1 with the most suspicious transcripts identified by sequencing (cf. Supplementary Methods). However, filter expressions can be modified or extended by the user at any step of the analysis. Our analysis of the CTL and VPA hepatocytes samples showed that technical artifacts affected a significant fraction of the reads (10.5% and 9.3%), with the largest contribution coming from transcript truncation (4.4% and 3.8%). This is followed by RTTS (3.8% and 3.4%) and internal priming (2.6% and 2.4%) (Fig. 1C).

### 3.2 LRTS transcriptome reconstruction is largely dependent on filtering criteria

Transcriptome reconstruction yields a high number of gene and transcript candidates. While a large fraction is supported by only few reads, or can be traced back to technical artifacts, the identified numbers by far exceed the number of annotated transcripts, with a great range of experimental evidence and biological properties. For any transcript-based downstream analysis, a well-tailored filtering strategy is crucial. To this end, we implemented a filtering query syntax that enables the user to efficiently implement arbitrarily complex filtering rules, to fit the specific requirements (Section 2). We demonstrate three different filtering strategies, here called *permissive*, *balanced*, and *strict*, suitable in different analysis situations. Filter queries for the strategies can be found in Supplementary Method S5. The effect of these filter strategies with respect to the novelty categories is shown in Supplementary Fig. S6.

With the 2 615 181 and 4 200 885 full-length reads of the CTL and VPA samples combined, we called 828 973 distinct intron chains as candidate transcripts, at 93 233 genomic loci (genes). For *permissive* filtering, we request at least two reads per transcript, and removing potential technical artifacts. This reduces the numbers to 21 447 genes, with 157 134 transcripts, of which 77.8% are not annotated in GENCODE. Since novel transcripts may require additional evidence, the *balanced* filtering additionally requires at least 7 reads (about one TPM) for transcripts not annotated in the reference, yielding 15 315 genes, with 57 698 transcripts, 49% of which are novel. However, many of these transcripts contribute less than 5% to the total read count of the gene, yielding complex gene models with up to 134 transcripts per gene even after balanced filtering. To simplify transcript analysis on gene level for downstream analysis, we suggest keeping only transcripts with a “substantial” contribution of at least 5% to the genes total, and at least 7 reads. This *strict* strategy yields 12 267 genes, with 21 582 transcripts, of which 32% are not found in GENCODE. Generally, stricter filtering yields higher fraction of known transcripts (full splice matches, FSMs), while novel combinations of existing splices sites (novel in catalog, NIC) as well as transcripts using novel splice sites (novel not in catalog, NNC) are reduced (Supplementary Fig. S6).

**Figure 2.**
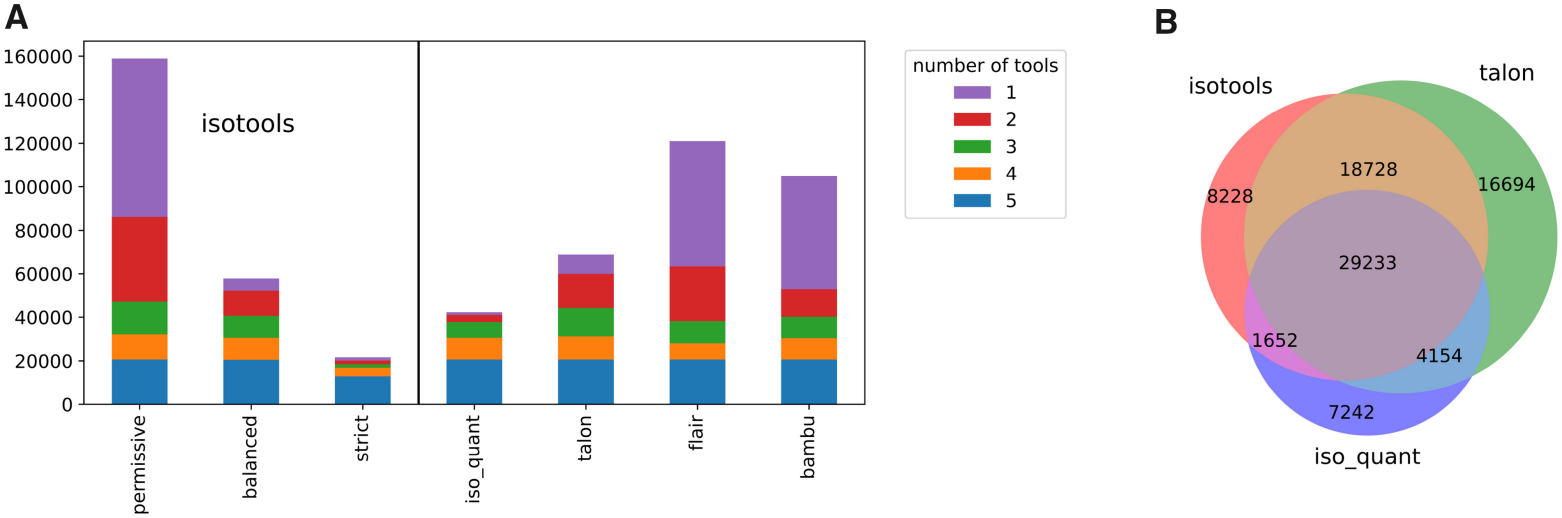
Comparison of recovered transcripts from IsoTools using different filter queries, with alternative tools for transcriptome reconstructions for long reads. IsoQuant, TALON, FLAIR, and Bambu were used with default filtering criteria. (A) Barplots (top) depict the number of reported transcripts per software and the color indicates the number of tools reporting a specific transcript. (B) Venn diagrams (bottom) depict the overlap between IsoTools with balanced filter, IsoQuant, and TALON.

We compared IsoTools transcriptome reconstruction with four other recently published tools for transcriptome reconstruction (IsoQuant [Prjibelski *et al.* 2023], TALON [Wyman *et al.* 2020], FLAIR [Tang *et al.* 2020], and Bambu [Chen *et al.* 2021]). Each tool was applied to the hepatocyte dataset with its default filtering criteria and IsoTools transcriptome reconstruction was run with varying filters. Bambu also reports reference transcripts not supported by long reads, these were manually filtered for subsequent comparisons. With permissive filtering, about half of the transcripts identified by IsoTools are shared with any of the other tools while the remaining 72 733 are uniquely recovered by IsoTools. This proportion of uniquely identified transcripts drops to less than 10% (5564 unique transcripts) with IsoTools balanced filtering strategy. With strict filtering, most of the transcripts are common with at least one other tool. While 20 642 transcripts are reported by all five tools, the total number of transcripts identified by each tool with default parameters varies substantially (Fig. 2 and Supplementary Fig. S7).

All tools except IsoQuant report genes with more than 100 transcripts. The high fraction of novel, not annotated transcripts demonstrates on the one hand the value and power of long reads for transcriptome reconstruction. On the other hand, the high number of transcripts observed may overwhelm and confound downstream analysis. Hence, transcriptome reconstruction is heavily dependent on carefully selected filtering criteria to extract qualified transcripts from the long reads. Alternatively, transcriptome reconstruction provided from other tools can be imported in IsoTools, using gtf files as interfaces. This can be used, e.g. to import different strategies for TSS recovery, and aids in identifying more robust transcript results.

### 3.3 Refined classification of novel transcripts facilitates biological interpretation

The discovery of novel transcripts by long reads presents both opportunity and uncertainty, as they may be functional variants overlooked by short-read technology or artifacts. To determine their nature, we refined the widely adopted classification scheme for novel transcripts introduced by SQUANTI (Tardaguila *et al.* 2018) with 19 subcategories that directly facilitate biological interpretation (Supplementary Method S6 and Supplementary Fig. S4).

In the primary hepatocyte LRTS, 73.5% and 74.5% of the reads (after filtering for technical artifacts) fully match known transcripts (FSMs) whereas the most prevalent category for novel transcripts are categorized as “novel combinations” of known splice junctions, affecting 9.9% and 9.3% of the reads, for CTL- and VPA-treated samples, respectively (Supplementary Fig. S5A). Often these combinations span a large genomic distance, and thus these novel transcripts are hardly detectable with short reads. Nonetheless, they can be highly expressed and thus might aid in complementing the reference annotation. One such novel transcript detected by this study is corresponding to the SPTBN1 gene (spectrin beta, non-erythrocytic 1), which combines the promoter of the transcript SPTBN1-201 with the PAS of SPTBN1-202 and SPTBN1-207, after sharing 30 intermediate exons with both transcript variants thus spanning a distance of 100 kb (Supplementary Fig. S5B). SPTBN1 has recently been assigned a role as a therapeutic target for nonalcoholic steatohepatitis and liver cancer and thus detecting further highly expressed transcripts of this gene is important. The novel transcript is supported by 3306 and 1620 reads (about 30% of all reads of that gene) and both ASEs affect the coding region, potentially yielding a novel protein sequence.

ES transcripts are among the next most frequent categories of novel transcripts; however, we found that a large part within this categories must be attributed to misalignment of short exons <30 bases, which are aligned to either of the neighboring exon boundaries. This issue affects up to 20% of the transcripts in these categories, and more than 50% of the highly covered transcripts with more than 50 IsoSeq reads (Supplementary Fig. S8), a technical artifact that can be approached at the alignment step (Sahlin and Mäkinen 2021). We used IsoTools filtering framework described above to select for longer skipped exons that contribute substantially to the total genes expression, and thus are likely functional. For example, we found a novel transcript of the gene PATL1, skipping the 102 bp exon 7, which is one of the major transcripts of the gene in both CTL- and VPA-treated hepatocytes (28.8% and 21.5%) (Supplementary Fig. S9). PATL1 is involved in mRNA degeneration, and the skipped exon overlaps the protein domain involved in RNA-binding. Junction coverage of short-read data from the same samples confirms ES of a similar proportion of transcripts. Notably, the ES event is also supported by short-read RNA-seq of human liver samples, demonstrating *in vivo* relevance.

### 3.4 IsoTools facilitates reliable analysis of alternative splicing

**Figure 3.**
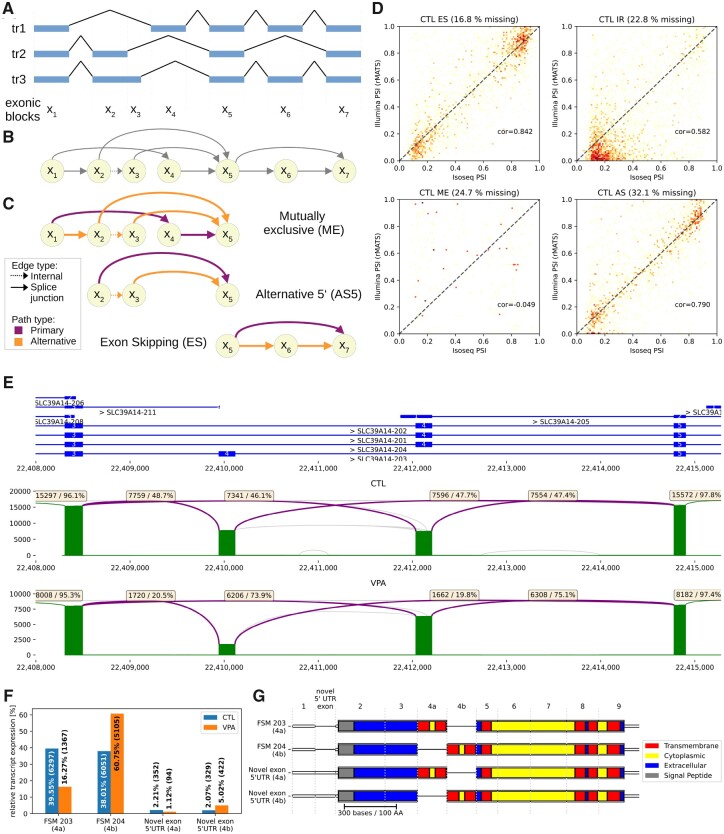
(A) Exemplary gene with three transcripts and (B) the corresponding segment graph. Solid arrows represent splice junctions, while the dotted arrow ($x_{2}$ to $x_{3}$) represents an internal edge. (C) Splice bubble decomposition of the segment graph identifies three ASEs and corresponding categories. The primary and alternative paths of the bubbles are depicted by purple and orange coloring of the edges, respectively. (D) Comparison of ASE quantification with IsoTools from IsoSeq and with rMATS from Illumina RNA-seq for the untreated hepatocytes, separately for the event classes: ES, IR, ME, and 5' and 3' alternative splice sites combined (AS). (E) Differential splicing of SLC39A14 ME exon 4 upon VPA treatment. Top track represents the reference annotation and bottom tracks show log scaled Sashimi plots of IsoSeq transcripts. Purple arcs represent splice junctions involved in the ASE, and are labeled with the total number and the fraction of supporting reads IsoSeq full-length reads. Other splice junctions which are supported by at least 1% of the transcripts are depicted as green arcs, and by less than 1% gray. (F) Relative contribution of major SLC39A14 transcripts to the total expression of the gene, for VPA and control samples. (G) Domain structure of major isoforms of SLC39A14 shows the two first transmembrane domains encoded in the ME exon 4.

We evaluated the ability of long-read coverage to quantify gene expression and compared it with RNA-seq (Supplementary Method S7 and Supplementary Fig. S10). Our analysis revealed good agreement in gene expression levels (*r* = 0.75 and 0.76 for VPA and CTL), confirming the suitability of long reads for expression quantification. However, we observed a reduced correlation on the transcript level (*r* = 0.42 and 0.43), which may on the one hand be attributed to challenges in transcriptome reconstruction using long reads, or bias and ambiguity from short-read transcript assignment on the other. To provide a quantification approach that is independent of the transcriptome reconstruction step, we developed a method to detect ASEs as bubbles in the segment graphs of gene models (Fig. 3A and B; cf. Section 2). This method enables us to break down the splicing complexity and isolate the individual events that differentiate transcripts.

ASEs are subdivided and classified according to the underlying molecular principles as ES, IR, 5' and 3' alternative splicing (5AS and 3AS), and ME exons (Fig. 3C). Relative expression of ASEs is quantified with the PSI, which is the fraction of reads supporting the alternative. Differential splicing events (DSEs) are identified with a two-proportions *z*-test when only two samples are under study as in our test case, or with a beta-binomial mixture likelihood test when comparing two groups of samples (see Section 2).

In total, we identified 3.797 IR, 2.713 ES, 1.526 3AS, 1.243 5AS, and 376 ME events in the primary human hepatocytes, covered by at least 100 reads over both VPA and CTL, where the alternative contributed at least 10% of the reads. 46.1% of these events are not annotated and thus challenging to quantify with short-read analysis tools, such as rMATS (Shen *et al.* 2014). To validate the LRTS quantification of both novel and known events, we provided rMATS with the identified events. Since the rMATS event definition is less flexible, not all events were taken into account, but 80.1% of the IsoTools events could be exported to rMATS, and were quantified with the short reads. We found high correlation of PSI values for ES (*r* = 0.84), 5AS (*r* = 0.8), 3AS (*r* = 0.74), and IR (*r* = 0.58), while quantification of more complex ME events was not correlated (*r* = −0.05) (Fig. 3D). This result suggests that our approach provides reliable ASE detection and quantification from LRTS, also for novel splicing events.

### 3.5 VPA induces DSEs in primary human hepatocytes on different levels of complexity

We applied the two-proportions *z*-test to identify DSEs between treated (VPA) and untreated (CTL) hepatocytes, and found 777 DSEs in 538 different genes at an FDR of 1%. These events include 26 ME, 253 ES, 90 5AS, 59 3AS, and 349 IR events, featuring different levels of complexity. Also for the differential events, short-read quantification with rMATS was in good agreement, with a Spearman correlation of 0.68 (Supplementary Fig. S12). We also found differential usage of 956 TSSs and 290 PASs between VPA and CTL. Supplementary Table S1 lists the differential splice events between VPA- and CTL-treated hepatocytes. These numbers suggest a widespread effect of VPA on the usage of TSS. Indeed, it has been shown that HDAC inhibitors (as well as DNMT inhibitors) specifically introduce cryptic TSSs in long terminal repeats (Brocks *et al.* 2017). This was shown for the HDAC inhibitor SAHA, a class I, II, and IV inhibitor, while VPA targets class I and II proteins.

While ME events are rare (3.3%), the most significant splicing event involves the ME exons 4A and 4B of SLC39A14 (Solute Carrier Family 39 Member 14), a metal cation transporter responsible for the uptake of trace elements such as zinc, iron, and manganese in the liver. Both versions, containing either exon 4A or 4B, yield functioning proteins, but the uptake kinetics vary substantially (Girijashanker *et al.* 2008). While we found both variants expressed at comparable level in the CTL sample, the proportion of 4B increased to 75% in the VPA-treated sample (Fig. 3E). A similar shift has been observed between normal and colorectal cancer samples (Thorsen *et al.* 2011). Notably, we found the same trend with rMATS using the short-read data when providing the events identified with IsoTools (42%–70% PSI of exon 4B), while rMATS alone did not identify the correct event. IsoTools identified this DSE with high confidence, highlighting the power of long-read sequencing for resolving complex splicing events. Furthermore, the long reads facilitate following up on functional analysis of the transcripts involved in the splicing event. IsoTools provides detailed annotation of the open-reading frames and domain structures of the isoforms (Supplementary Method S8), such as predicting the effects of the splicing event on protein function. For the ME event in SLC39A14, all major transcripts representing the two variants of the ME event are full-length transcripts. The two major transcripts, which together make up 77% of the gene’s expression, correspond to reference transcripts 203 (with exon 4A) and 204 (with exon 4B). However, two additional transcripts are expressed, both containing a novel exon in the 5'-UTR, and show the same effect of VPA on the ME exon as the canonical forms (Fig. 3F). Annotating and visualizing the InterProScan domain structure (Blum *et al.* 2021) confirm that exons 4A and 4B are functionally similar, both encoding the first two transmembrane domains (Fig. 3G).

ES events account for 32.6% of differential splicing. The most significant differential ES event affects increased inclusion of exon 32 of the FN1 gene (fibronectin 1; *q*-value = 3.55*E*−111, PSI 9% in CTL and 15% in VPA). Again, rMATS analysis of short-read RNA-seq confirmed this differential event (PSI in CTL 12% and VPA 18%). FN1 is one of the first genes for which alternative splicing was described, and regulation and functional effects of splicing in FN1 have been studied extensively (Kornblihtt *et al.* 1984). The particular exon subject to the skipping event is referred to as extra domain A (EDA), one of two extra domains of this gene, and inclusion is regulated by serine/arginine-rich splicing factors SRSF1 and SRSF3. While the extra domains are essential for normal development, elevated inclusion of EDA is associated with several diseases, including psoriasis, rheumatoid arthritis, diabetes, and cancer (White *et al.* 2008). Previous studies observed similar effects on splicing of FN1 triggered by HDAC inhibitor sodium butyrate (NaB), but in this case resulting in elevated inclusion of exon 23, which is extra domain B (Hnilicová *et al.* 2011). Aberrant splicing of FN1 may be related to hepatotoxic effects, as downregulated or dysfunctional SRSF3, and subsequent aberrant splicing of its targets including elevated EDA-Fn1, has been associated with liver disease (Kumar *et al.* 2019). We thus conclude that the implemented LRTS workflow reliably identifies DSEs on different levels of complexity.

## 4 Discussion

Here, we presented IsoTools, a flexible and powerful Python framework for the analysis of LRTS data. It provides data structures to search, access, and filter the transcripts, as well as functionality to compute QC metrics, to compare and annotate the transcripts with reference annotations, to integrate data from several LRTS experiments, to quantify transcript expression levels based solely on LRTS, to perform statistical analysis for differential splicing, and to export of the data in several output formats. In addition, the tool facilitates the depiction of summary statistics as well as complex splicing models of individual genes and transcripts. IsoTools stands out with its unparalleled flexibility, thanks to its modular design and interfaces for custom metrics and algorithms. This makes it a highly adaptable solution for LRTS data analysis, particularly for advanced users seeking to tackle novel use cases, such as single cell LRTS analysis. The tool’s ability to import and export transcriptomes in gtf format not only provides added convenience, but also ensures compatibility with other tools and facilitates integration into existing workflows. With the ability to import transcriptomes from different sources, users can also easily compare and validate the results from different pipelines to ensure the accuracy of their analysis. The modular design of IsoTools represents a significant departure from traditional, rigid data analysis tools. This innovative approach allows users to fully customize the tool and explore new applications, making IsoTools a truly unique and valuable resource for LRTS data analysis.

To characterize novel transcripts, we introduce a fine grade biologically motivated classification scheme, refining the established technically defined classes. The categories facilitate direct interpretation of differences between samples, and may hint toward specific disturbed splicing mechanisms, such as introduced by SF3B1 hotspot mutations, which specifically result in shifted in 3' splice sites (Darman *et al.* 2015). By far, the most common category of novel transcripts was “novel combinations of known splicing events.” Often, these combinations are separated by several kilobases, and thus cannot be identified with short-read sequencing.

We propose a graph-based approach to identify and classify alternative splicing, based on bubbles in the segment graph. This definition yields binary events, which are classified by event type and quantified with PSI values. These events provide the basis for statistical tests for the detection of differential splicing, either between two samples or groups of samples. This approach is fundamentally different from differential expression analysis on transcript level, for which the well-established framework based on negative binomial generalized linear models (Robinson *et al.* 2010, Love *et al.* 2014) can be applied, also with LRTS data (Reese and Mortazavi 2020). However, identification of DSEs has several advantages over differential transcript expression (DTE). While DTE may be a combination of differential regulation on gene level and differential splicing, DSE focuses on local splicing regulation, and is independent from gene expression levels. Further, DSE can distinguish several related or independent splicing events on the same gene. As the individual events can be classified, DSE facilitates categorization of differential splicing. Last, DSE aggregates statistical power from all transcripts covering the event. Even transcripts affected by technical artifacts, such as RTTS and fragmentation, may still provide useful information for event-level analysis. On transcript level, these artifacts would result in distinct transcripts, and thus further increase the complexity and disturb the analysis if not filtered out. Hence, to interpret and validate the effects of differential splicing, DSE analysis yields more concise results. While differential splicing analysis is also possible with short-read RNA-seq, long-read technology offers several advantages for this task. With long reads complex splicing events can be resolved and quantified with high confidence, which can be difficult using short reads. In addition, long reads do not suffer from alignment ambiguity and reduce the dependence on reference annotations, which is particularly important when dealing with novel transcripts and splice junctions. Moreover, long-read sequencing identifies the full-length transcripts, enabling the functional interpretation of splicing events by inferring nonsense-mediated decay and annotating protein domains and thus provides a more complete understanding of the functional impact of alternative splicing.

Much like gene expression, alternative splicing is subject to biological variability within samples and groups of samples of the same condition. Statistical tests that compare individual samples, such as the two-proportions *z*-test and the likelihood ratio test with binomial model implemented here, neglect this variability, making the analysis prone to false positive results. Thus, the third implemented test, the likelihood ratio test with beta-binomial model, facilitates the comparison of groups of samples. The biological variability within the groups is estimated from the data and taken into account. While this approach promises to be more robust, it depends on replicates and thus could not be demonstrated with our data; however, it is demonstrated with data from ENCODE in the differential splicing tutorial which is part of IsoTools online documentation. To date, LRTS datasets with biological replicates are the exception, but continuously falling sequencing cost, higher throughput in combination with sample multiplexing, as well as better software facilitating additional applications will improve the cost benefit ratio of biological replicates.

In primary human hepatocytes, IsoTools identified aberrant splicing events in different categories, caused by the HDAC inhibitor VPA. The role of HDACs in modulating alternative splicing has recently been emphasized by investigating the role of histone marks in the choice of splice sites and regulation of splicing (Rahhal and Seto 2019). We observed changes in splicing after VPA treatment on all levels of complexity but most prominently with usage of ME exons, ES, and alternative TSS events, and we validated these events by short-read RNA-seq of the same samples. While not yet reported after VPA treatment, many of the identified differential events have been observed to be triggered also by other HDAC inhibitors in comparable models, demonstrating the ability of LRTS to detect differential splicing between samples.

IsoTools is simple to install, flexible, versatile, and easy to use. It offers novel functionality, including expression quantification and differential splicing analysis, extending the range of potential applications for LRTS. Its main strength comes from the open and modular framework design, facilitating exploitative analysis and development of specific use cases. For starters, the extensive documentation contains several example workflows, covering relevant use cases which can simply be adapted by the user. To realize standardized workflows, there is also a command line interface, providing some of the functionality without the need to write python code. We demonstrated the utility of our tool by analyzing LRTS data from hepatocytes treated with VPA, and identified novel and DSEs, of which several are expressed also in human liver samples and are thus likely relevant *in vivo*.

## Supplementary Material

btad364_Supplementary_Data

## Data Availability

The primary human hepatocyte LRTS data is available from ENA under accession number PRJEB46194.
