# Supplementary material for: IsoTools: a flexible workflow for long-read transcriptome sequencing analysis
Source: Bioinformatics. 2023 Jun 2;39(6):btad364. doi: 10.1093/bioinformatics/btad364 (PMC10287928; doi:10.1093/bioinformatics/btad364)
Supplement: btad364_Supplementary_Data [file btad364_supplementary_data.zip › SupplementaryInformation.pdf]

IsoTools – a flexible workflow for long-read transcriptome sequencing analysis
Supplementary Materials

Matthias Lienhard[1,*], Twan van den Beucken[2], Bernd Timmermann[3], Myriam Hochradel[3], Stefan Boerno[3], Florian Caiment[2], Martin Vingron[1], and Ralf Herwig[1,*]

[1]*Max Planck Institute for Molecular Genetics, Department of Computational Biology, Ihnestraße 63-73, GERMANY*
[2]*Maastricht University, Department of Toxicogenomics, Universiteitssingel 40, 6229ER Maastricht, NETHERLANDS*
[3]*Max Planck Institute for Molecular Genetics, Sequencing core unit, Ihnestraße 63-73, 14195 Berlin, GERMANY*
[*]*To whom correspondence should be addressed.*
ML: Tel: +4930 8413 1675; Email: lienhard@molgen.mpg.de
RH: Tel: +4930 8413 1126; Fax: +4930 8413 1152; Email: herwig@molgen.mpg.de

Part I
Supplementary Methods

1 PacBio IsoSeq Sequencing

For each time point and condition, we prepared triplicate cDNA samples. For library preparation, all VPA treated samples as well as the control samples were pooled. The libraries were sequenced on the PacBio Sequel II platform, using one 8M SMRT cells for each pool. 300 ng each of total RNA isolates of triplicates of 4 control and 4 treatment conditions were used for cDNA generation following PacBio's isoseq library preparation protocol. Therefor, after confirming the integrity of each sample using Agilent Bioanalyser, we used the NEBNext®Single Cell/Low Input cDNA Synthesis & Amplification Module.

After confirming sample integrity using Agilent Bioanalyser, 300 ng RNA from each triplicate sample was incubated with NEBNext Single Cell RT Primer Mix for 5 min at 70 °C. Afterwards, Single Cell RT buffer and Single Cell RT enzyme Mix were added and incubated for 75 min at 42 °C. Then, template switching oligo was added, followed by 15 min of incubation at 42 °C. cDNA was cleaned up using 1:1 Pronex beads followed by two washing steps with 200 µL 80% ethanol and elution of the sample with EB. After addition of NEBNext Single Cell cDNA PCR master mix, NEBNext primer and the Iso-Seq Express cDNA PCR primer, cDNA was amplified in 12 cycles with 3 min elongation time. We targeted for standard transcript length of 2kb using 86 µL of Pronex beads for cleanup.

For sequencing, all control and VPA-treated samples were pooled equimolarly and PacBio IsoSeq libraries were prepared using the Express TPK 2.0 kit, including damage repair, end repair and A-tailing steps. Next, overhang adapters were ligated and the resulting libraries were cleaned up using 1:1 ProNex beads. Agilent bioanalyser assessment reported an average library insert sizes of 4,222 bp for CTL and 4,336 bp for VPA treated samples, respectively. Sequencing complexes were generated using Sequel II Binding Kit 2.0, Sequencing Primer v4.0, and Sequel II Polymerase 2.0, and then purified using ProNex beads.

Libraries for the pooled CTL and VPA treated samples were loaded individually together with PacBio internal control libraries on two Sequel II SMRT cells (diffusion loading). Sequencing was conducted with 30 h movie time, yielding 660.7 GB (CTL) and 460.1 GB (VPA) of total sequencing data. This resulted in 6,720,864 and 3,926,050 polymerase reads, with an average length of 98,305 and 117,202 bases.

2 PacBio IsoSeq Pre-processing

IsoSeq subreads were processed using the Iso-Seq v3.4 bioinformatic pipeline with recommended parameters. In brief, we used the `ccs` tool to call circular consensus sequences, lima to remove primers and adapters, and `isoseq refine` to filter out reads not featuring poly-A sequences. This resulted in 2,643,404 and 4,265,020 "full length non chimeric" (flnc) HiFi poly-A reads, with an average length of 3,636 and 3,880 bases for the VPA and CTL samples, respectively. 96.6% and 95.5% of these HiFi reads have an error rate of less than 1%, according to base quality values. The flnc reads were directly (without an additional clustering step) aligned to the human genome GRCh38.p13, obtained from the GENCODE website (Frankish *et al.*, 2019), by calling minimap2(Li, 2018) with the `pbmm2 align --preset ISOSEQ` command. 2,611,571 and 4,196,197 of the reads could be aligned in one consecutive alignment, 30,561 and 66,293 were split in two or more parts, 1,272 and 2,530 were not aligned. All further analysis steps were performed with the IsoTools package, as described here and in the respective results sections. For these samples, the analysis including data import, computation of quality control metrics, characterization of novel transcripts, and differential analysis took about 67 minutes on a single CPU core, using a maximum of 20 GB RAM.

3 Saturation Analysis

IsoTools provides a model-based approach to estimating the required sequencing depth, complementing the widely adopted approach of rarefaction analysis. This method models the probability of observing a transcript with at least r reads based on the sequencing depth n (total number of long reads) and the expression level p (in TPM) of the transcript. While two copies of a transcript is the preset recording threshold of the PacBio IsoSeq clustering pipeline, it may be reasonable to reduce or increase this threshold. With IsoTools saturation analysis, the user can choose the number of reads r based on their specific use case. For instance, when the goal is to discover rare transcripts, a lower coverage threshold may be sufficient. In such cases, even a single read may provide compelling evidence, which can then be validated through experimental methods. On the other hand, when conducting differential analysis between samples, a higher coverage threshold is required. Two reads may not provide enough evidence to detect any significant differences in Figure expression levels between samples. Figure S2 shows the result of the model for 1, 2, 5, and 20 reads.

The model can be used for experimental planning or retrospective assessment of transcript coverage. For example, the method can answer questions such as the required sequencing depth to cover 90% of all 1 TPM transcripts with at least one read or the probability of covering a 1 TPM transcript with at least one read given a specific sequencing depth.

4 Definition of Artefact Tags

To facilitate control for technical artefacts, we consider three confounding effects known to occur in LRTS data (Tardaguila *et al.*, 2018):

- Genomic stretches of adenosines may be bound by the poly-A primer, inducing sequencing of genomic templates, which are not spliced and thus yield a single exon transcript in

LRTS. This effect is called **internal priming**.

- The ability of the reverse transcriptase to switch between templates (**RTTS**) is exploited to anneal the primers during the SMRT sequencing. However, this switching can also occur unintended within or between templates, resulting in incomplete or chimeric templates. This template switching preferably occurs at short direct repeats.
- Transcripts may be **truncated** during library preparation, resulting in transcript fragments. Due to poly-A priming, 3' fragments are unlikely to be sequenced, but 5' fragments yield incomplete transcripts with apparent novel transcription start sites.

To derive default definitions to tag these artifacts, we compared related metrics from the most credible GENCODE transcript with support level 1 (assuming they are genuine) to the most suspicious isoforms identified by LRTS. However, all filter expressions may be adapted or extended by the user.

To identify internal priming, we monitor the fraction of adenosines in the genomic sequence 30 bases downstream of the transcript. For the highly credible (support level 1) GENCODE isoforms we find a downstream adenosine content distributed with a single mode around 25% adenosines. These were compared to single exon genes that do not overlap any annotated gene, presumably containing a large fraction of internal priming artefacts. For this group, we observed a bimodal distribution, with the second mode at 70% adenosines (Suppl. Fig. S3B). Based on this observation, we chose a threshold of 50% downstream adenosienes of monoexonic transcripts to mark internal priming. Accordingly, the default expression for the "INTERNAL_PRIMING" tag is: `len(exons)==1 and downstream_A_content and downstream_A_content > 0.5`.

To select for putative RTTS sites, we screen for introns without reference support where both donor and acceptor sites are within reference exons, and which do not feature a canonical splice site. At the boundaries of these introns, we compute the length of direct exact repeats and compare it to the repeat length at regular introns of the high confidence GENCODE isoforms (Suppl. Fig. S3C). While the putative RTTS sites feature a slightly longer average repeat length of 5 bases compared to 2 for the regular introns, this difference does not allow to define a threshold that would separate the majority of putative RTTS transcripts from the credible transcripts. In contrast to other tools, which rely on this metric, we tag transcripts as RTTS if they have a non-canonical, non-reference intron, e.g. both donor and acceptor are not annotated and the sequence at the splice site is not 'GT-AG'. This translates to the expression

```
noncanonical_splicing is not None
and novel_splice_sites is not None
and any(2*i in novel_splice_sites
        and 2*i+1 in novel_splice_sites
        for i,_ in noncanonical_splicing)
```

for the "RTTS" tag.

We tag all transcripts that start or end within an internal exon of another transcript, but share all other splice sites with this transcript, as a potential fragment. This is checked by the expression

```
fragments and any("novel exonic" in a or "fragment" in a
                  for a in annotation[1])
```

to set the FRAGMENT tag. In order to assess these potential truncations, we took additional evidence from publicly available CAGE data into consideration. To this end, we downloaded CAGE TSS peaks of HepG2 cells from ENCODE (Davis *et al.*, 2018). 11.2% of the TSS of potential 5'fragments overlap CAGE peaks in HepG2 cells from ENCODE, compared to 76.8% of all transcripts expressed with at least 2 reads that correspond to high confidence GENCODE transcripts, suggesting that many of the fragments are indeed truncated transcripts.

5 Definition of Filter Queries

To form a query string for the export functions, the tags are combined in logical expressions. This feature is demonstrated by the definition of the three filtering strategies:

- The *permissive* filtering strategy selects reference transcripts, as well as novel transcripts which are not tagged as artifacts, as long as they are supported by two or more reads.
- The *balanced* filtering strategy requires at least 5 reads for novel transcripts. This can be realized with a custom filter tag HIGH_COVER.
- The *strict* filtering requests at least 5 reads for all transcripts (`min_coverage` parameter), and at least 5% contribution to the genes total, ensured by the predefined tag SUBSTANTIAL.

The code block below lists the corresponding filter definitions:

```
# add the custom filter
isoseq.add_filter( "HIGH_COVER",'g.coverage.sum(0)[trid]>= 7',
  context='transcript')

permissive={
  "query": "FSM or not ( RTTS or INTERNAL_PRIMING or FRAGMENT )",
  "min_coverage": 2
}
balanced={'query':
  'FSM or (HIGH_COVER and not (RTTS or FRAGMENT or INTERNAL_PRIMING))',
  'min_coverage':2
}
strict={'query':
  'SUBSTANTIAL and (FSM or not (RTTS or FRAGMENT or INTERNAL_PRIMING))',
  'min_coverage':7
}

```

6 Categorization of Novel Transcripts

To categorize novel transcripts, Sqanti introduced a broad classification scheme that has been widely adopted. Novel transcripts are compared to the reference transcriptome, and categorized as full splice matches (FSM), incomplete splice matches (ISM), novel in catalog (NIC), and novel not in catalog (NNC). We have refined the classification by adding 19 different subcategories that provide deeper biological insight and facilitate the understanding of underlying biological mechanisms:

- ISMs correspond to fragments of reference transcripts, and IsoTools distinguishes ”5’ fragments”, ”3’ fragments” and ”mono-exons”.
- NIC transcripts are sub-classified in ”exon skipping”, ”intron retention”, ”novel combinations” of known splice junctions and other ”novel junctions”, e.g. both splice sites are annotated, but not used by the same junction according to the reference. Further, if the first or last exon shares its splice site with an internal reference exon, the transcript is classified as ”novel exonic TSS” or ”novel exonic PAS”.

- NNC contains transcripts with "novel 5' splice sites" (splice donors), "novel 3' splice sites" (splice acceptors), or "novel exons", not overlapping any reference exon. If a novel exon is the first/last exon of the transcript, it is classified as "novel intronic TSS/PAS". If a transcript includes splice sites from more than one reference gene it is classified as "readthrough fusion".
- Finally, transcripts not overlapping any splice junctions from the reference are classified as novel genes. Here, we follow the subclassification of Sqanti and distinguish "genic genomic" (implying exonic overlap with a reference gene), "intronic", "antisense" and "intergenic" transcripts.

While the Sqanti categories are mutually exclusive, a transcript in IsoTools may be assigned to several subcategories. Suppl. Fig. S4 provides prototypical examples for all different subcategories. To find these subcategories we compare the exon structure of an LRTS transcript to the segment graph of the reference gene. This implementation facilitates the definition of a set of rules to identify the relevant subcategories. As the subcategories are biologically meaningful, they may suggest biological processes involved in producing the novel transcripts (Fig. S5A). The subcategories are accessible from IsoTools query framework described above, facilitating identification of interesting examples for specific subcategories.

7 Long Reads Transcript Level Expression Quantification

IsoTools offers transcript quantification as well as splice event quantification by counting the respective long read coverage. Previous approaches used LRTS mainly for transcriptome reconstruction and applied parallel sequencing of the samples under study with RNA-seq for quantifying the detected transcripts (hybrid sequencing). However, recent advances with the new Sequel II system in terms of number of sequenced molecules as well as throughput raised the question to which extent direct quantitative interpretation of the transcript counts is possible. We have challenged this question by comparing the LRTS transcript quantification with RNA-seq quantification derived from the same samples. After basic normalization for sequencing depth, both hepatocyte samples had a similar distribution of read counts over transcripts (Suppl. Fig. S11). Consistent with recent results, we found good agreement between RNA-seq gene expression levels and LRTS coverage ($r = 0.756$ and $r = 0.765$ for CTL and VPA samples respectively), underlining the quantification performance (Fig. S10A). On transcript transcript level, however, the correlation dropped considerably ($r = 0.421$ and $r = 0.427$), also confirming previous results (Fig. S10B). A plausible explanation for the reduced accordance on transcript level is the uncertainty in assigning ambiguous short RNA-seq reads, and the errors introduced due to violated assumptions of uniform read coverage and imperfect reference model on the one hand but also insufficiency of LRTS sequence coverage. Furthermore, to investigate a potential transcript length bias, we analyzed the deviation of LRTS-derived expression levels from RNA-seq derived expression levels, in dependence on the transcript length. For transcripts $> 3,000$ bp, this deviation was constant, reflecting good agreement between LRTS and RNA-seq. However, for shorter transcripts, LRTS yields lower expression levels, suggesting a depletion of short transcripts within our data (Fig. S10C-D). This finding is in line with the comparison of the transcript length distribution to the reference annotation (cf. Fig. S3A).

8 Functional annotation

To facilitate functional analysis, IsoTools performs several steps. First, it annotates the longest open reading frame (ORF) for each transcript sequence, starting with "ATG" and ending with the next in-frame stop codon ("TAA", "TAG", or "TGA"). This allows the prediction of whether

a transcript would be degraded by nonsense-mediated decay (NMD) by applying the 55 base rule Nagy and Maquat (1998). Specifically, if there is at least one splice site upstream of the stop codon and the distance between the stop codon and the last upstream splice junction is less than 55 bases, the transcript is predicted to be targeted by NMD. Next, the resulting ORF is translated to a protein sequence, and functional protein domains are annotated. IsoTools incorporates three different types of sources for protein domain annotation. First, it uses annotation-based domains from UniProt UCSC genome browser tracks [1]. This is the fastest approach, but it may not cover novel exonic regions or splice junctions. Second, it uses alignment-based domains by aligning domain models to the protein sequences using PyHMMER. Models and metadata for Pfam domains are available for download on the InterPro website [2]. Lastly, domains can be obtained by employing the EBI InterPro REST API. By combining these sources, IsoTools provides a comprehensive annotation of protein domains that can aid in functional analysis of identified transcripts.

References

Davis, C. A. *et al.* (2018). The encyclopedia of dna elements (encode): data portal update. *Nucleic acids research*, **46**(D1), D794–D801.

Frankish, A. *et al.* (2019). Gencode reference annotation for the human and mouse genomes. *Nucleic acids research*, **47**(D1), D766–D773.

Li, H. (2018). Minimap2: pairwise alignment for nucleotide sequences. *Bioinformatics*, **34**(18), 3094–3100.

Nagy, E. and Maquat, L. E. (1998). A rule for termination-codon position within intron-containing genes: when nonsense affects rna abundance. *Trends in biochemical sciences*, **23**(6), 198–199.

Tardaguila, M. *et al.* (2018). SQANTI: extensive characterization of long-read transcript sequences for quality control in full-length transcriptome identification and quantification. *Genome Res.*

[1]https://genome.ucsc.edu/cgi-bin/hgTables
[2]https://www.ebi.ac.uk/interpro/download/Pfam/

Part II
Supplementary Figures

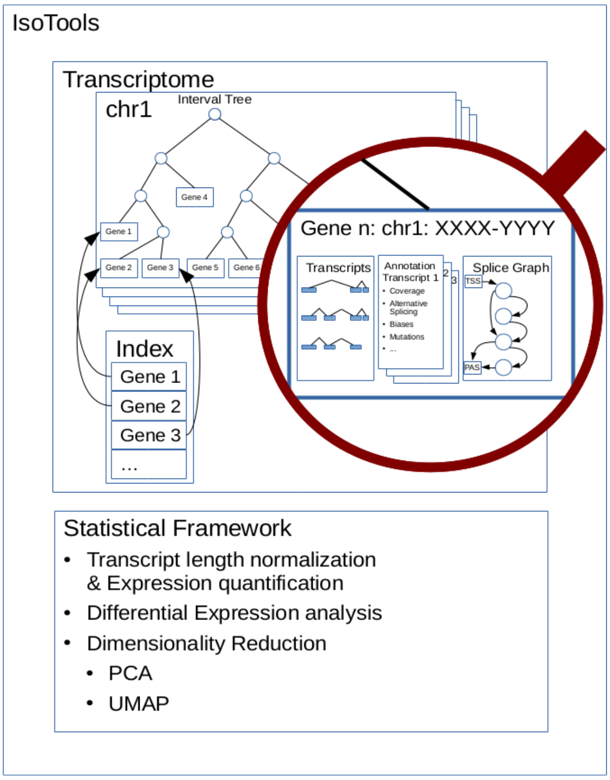

Figure S1: The internal data structure of the IsoTools framework

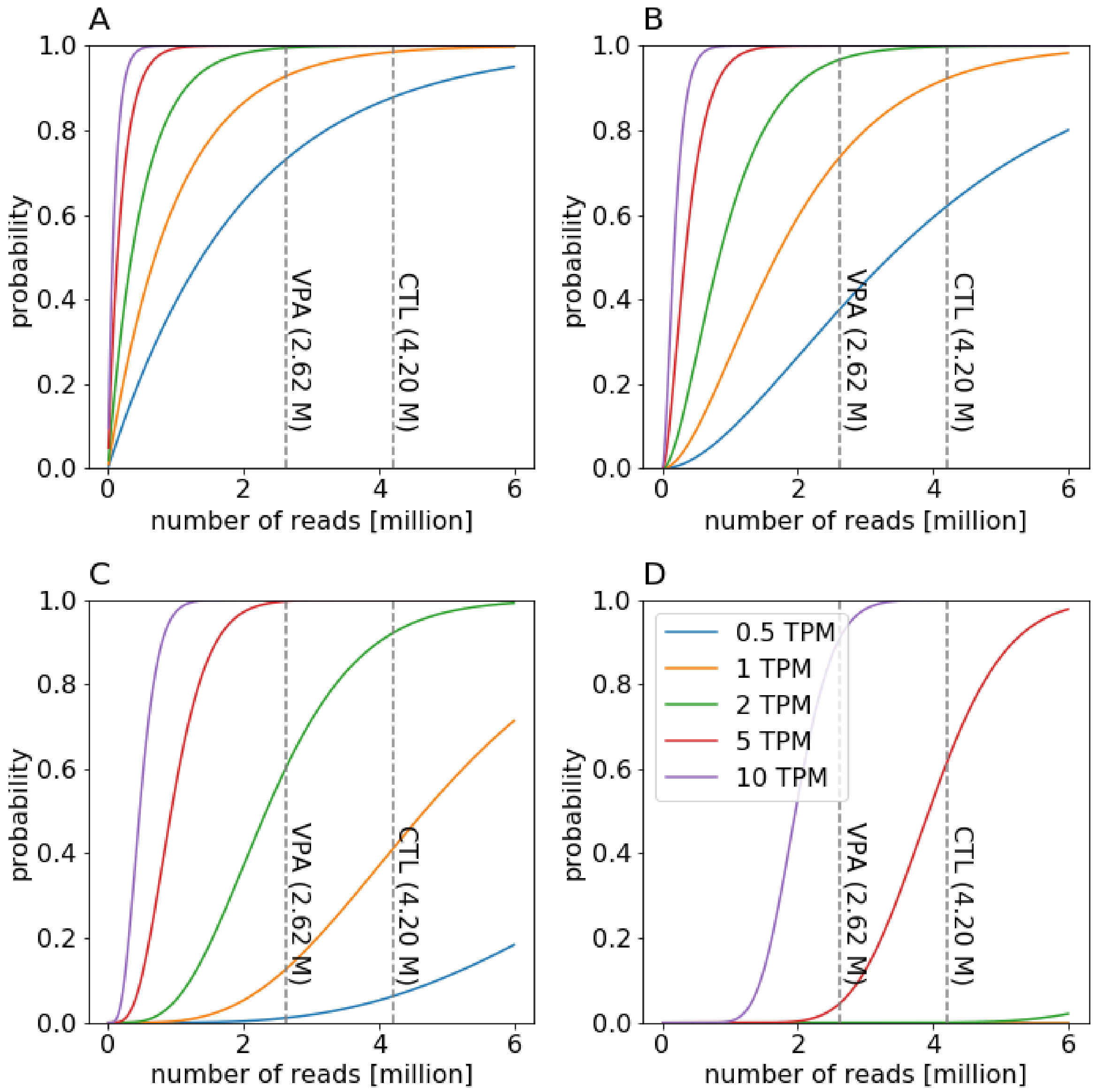

Figure S2: Saturation analysis. Subpanels show the modeled probability of observing a transcript depending on the cellular concentration from 0.5 (blue line) to 10 TPM (purple line) and sequencing depth at detection threshold of 1 read (A), 2 reads (B), 5 reads (C) and 20 reads (D). Dashed horizontal lines represent the sequencing depth of the VPA and CTL IsoSeq samples.

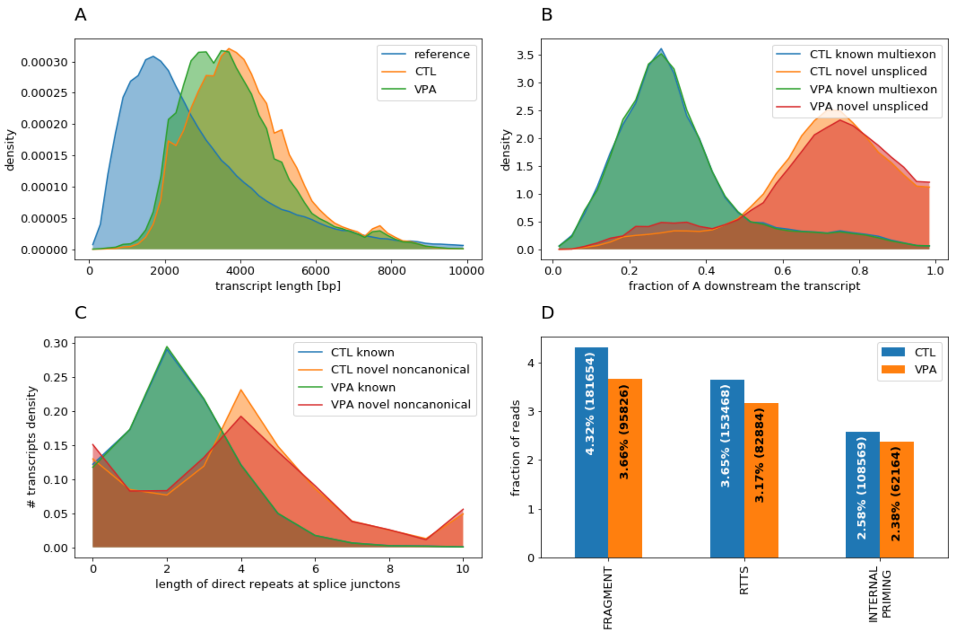

Figure S3: Quality control metrics. A) Read length distribution compared to level 1 GENCODE transcripts. B) A content downstream of novel unspliced transcripts and reference matching multi-exon transcripts. C) Direct repeat length at intron boundaries of GENCODE transcripts and novel non-canonical splice junctions. D) Fraction of reads affected by one of the three artifacts.

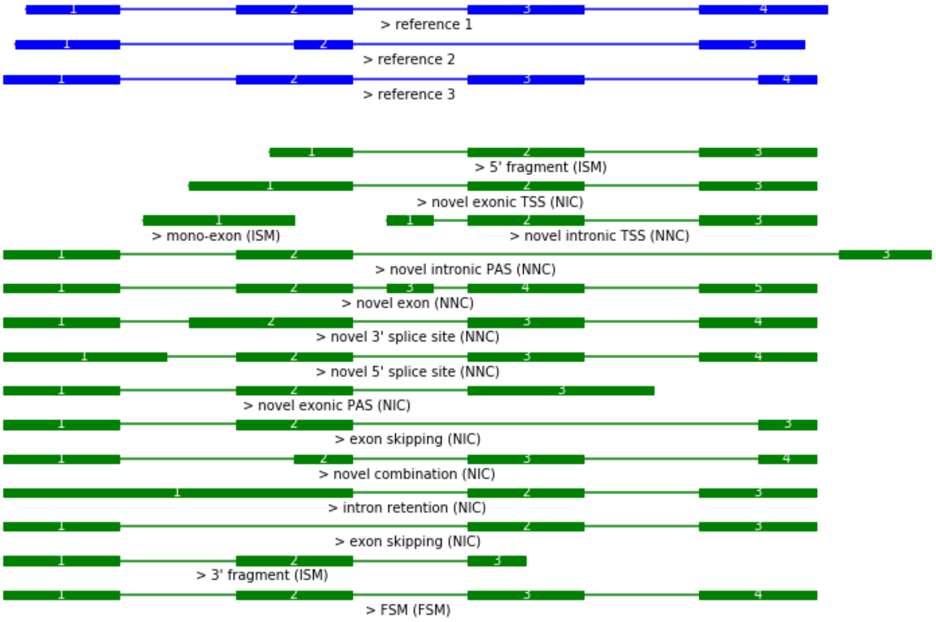

Figure S4: Prototypical examples for novel transcript classes. Reference transcripts are depicted in blue, and classified novel transcripts in green. Labels indicate the novelty class, with the Sqanti classification in brackets.

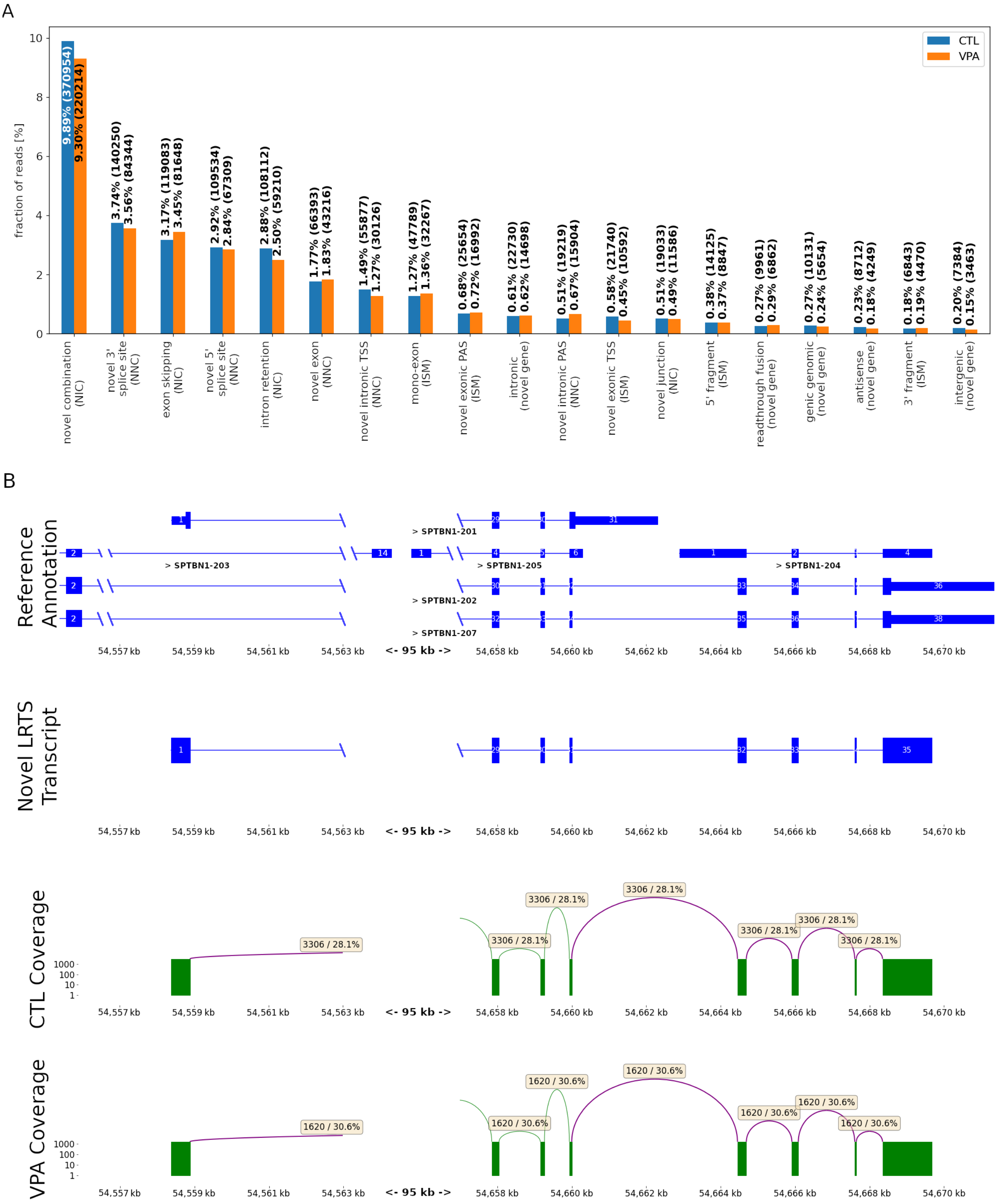

Figure S5: **A** Alternative splicing classification of the IsoSeq reads not fully matching reference annotation for CTL and VPA treated hepatocyte samples. **B** The novel transcript of the SPTBN1 gene is a combination from the TSS (left) of transcript SPTBN1-201 (first line in the top track) and the polyA site (right) of transcripts SPTBN1-202 and SPTBN1-207 (third and forth lines in the top tracks). The top gene track depicts the reference transcripts, and below the novel IsoSeq transcript. Coding regions in the reference transcripts are represented by broader blocks. The Sashimi plots in the bottom represent the coverage of this transcript for CTL and VPA hepatocyte samples respectively.

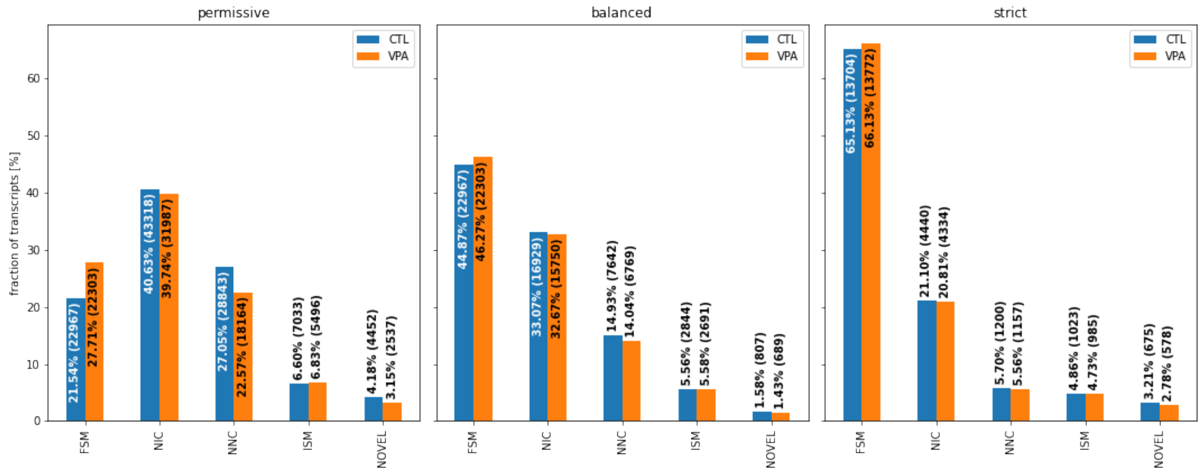

Figure S6: Fraction of transcripts in the different novelty categories passing the permissive, balanced, and strict filter criteria (f.l.t.r.)

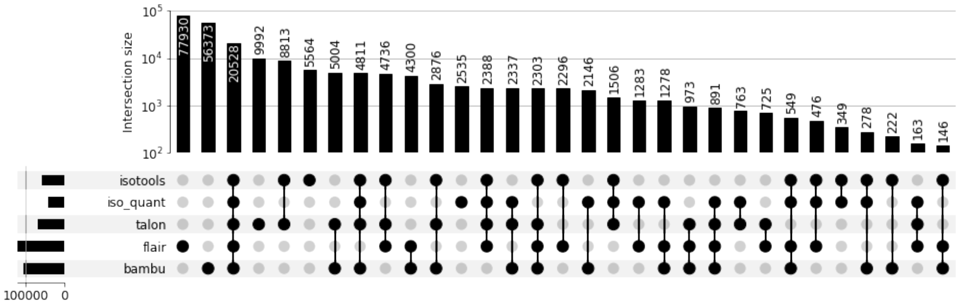

Figure S7: Upset plot of the recovered transcripts from IsoTools with balanced filtering, and the four alternative tools IsoQuant, TALON, FLAIR, and Bambu

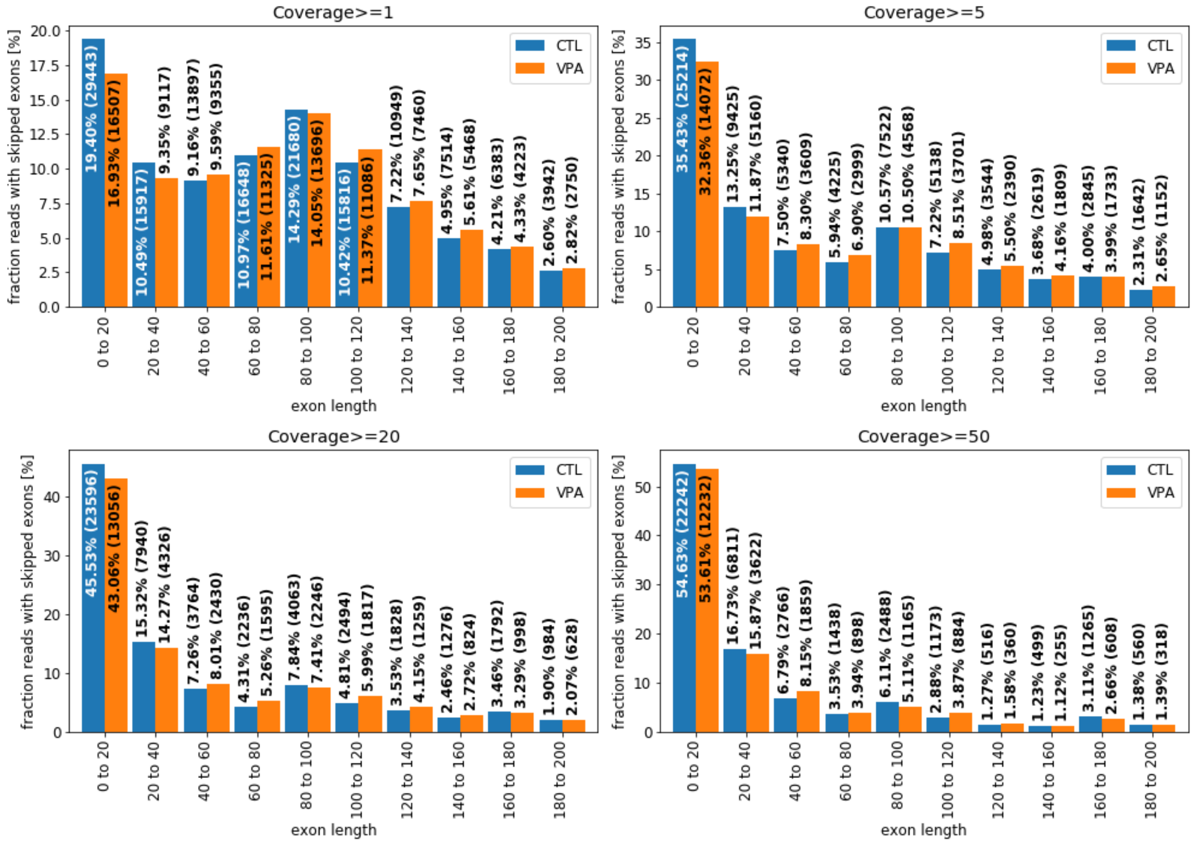

Figure S8: Histogram of novel exon skipping transcripts for different ranges of exon lengths. Short exons are not correctly aligned by the alignment tool, resulting in the detection of false skipped exons.

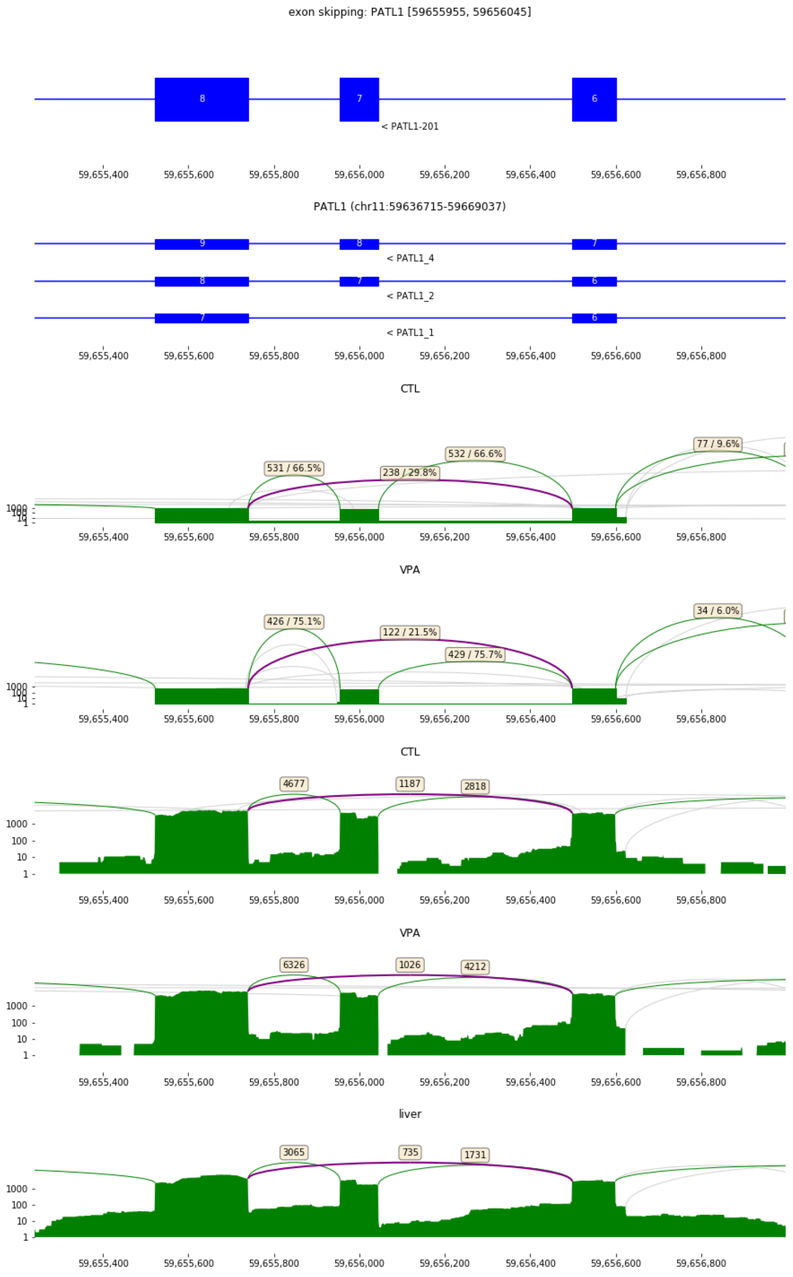

Figure S9: The novel isoform of PATL1 skipps exon 7 of the GENCODE annotation (top track). Relevant isoforms detected by IsoSeq are depicted in the second track. The following two Sashimi plots depict the IsoSeq coverage for the hepatocytes (CTL and VPA), and the bottom three Sashimi plots Illumina RNA-Seq coverage. The exon skipping junction is depicted in purple, other highly covered junctions in green, and poorly covered junctions in grey.

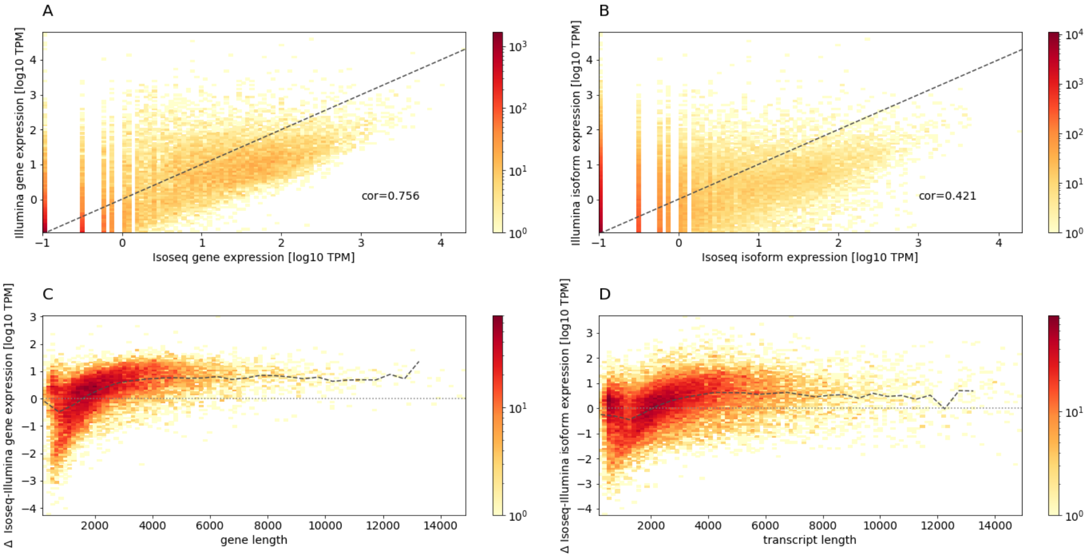

Figure S10: Comparison of expression levels derived from Illumina RNA-seq and IsoSeq on gene level **A** and transcript transcript level **B**. RNA-seq vs IsoSeq expression log10 difference depending on transcript length on gene level **C** and transcript level **D**. Dashed line represents the binned average.

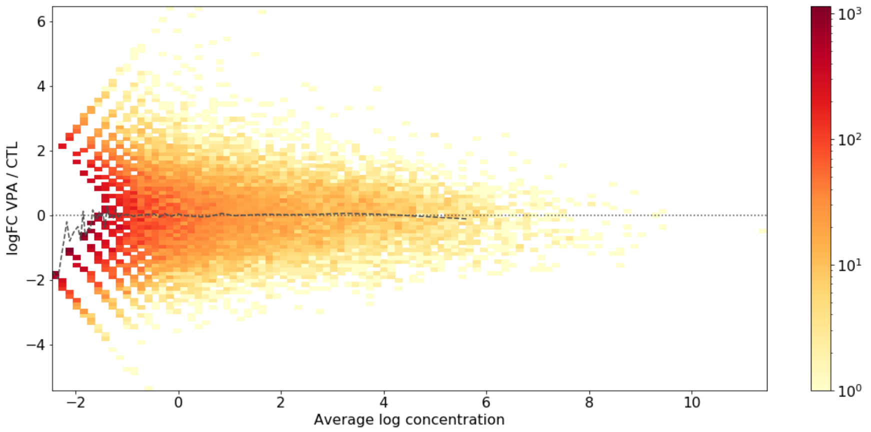

Figure S11: MA plot of upper quantile normalized IsoSeq transcript read counts in VPA vs CTL treated hepatocytes. Dashed line represent the binned average of logFCs, within equally sized bins of 500 transcripts.

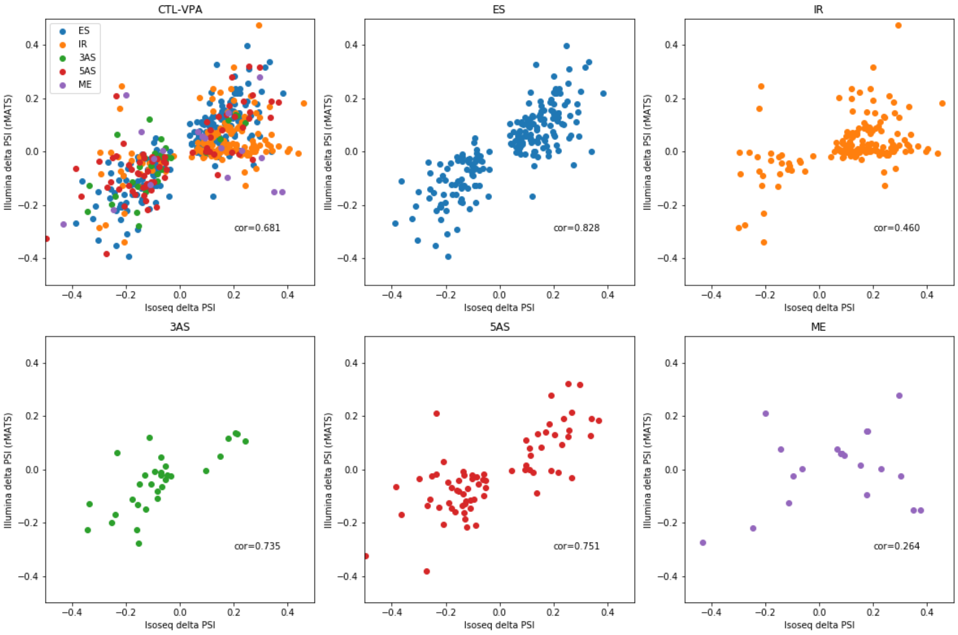

Figure S12: Comparison PSI differences between VPA treated and control samples for differential ASE (two-proportions z-test $FDR < .01$), quantified by IsoTools from LRTS and rMATS from short read RNAseq, for all events and the splicing event classes individually.

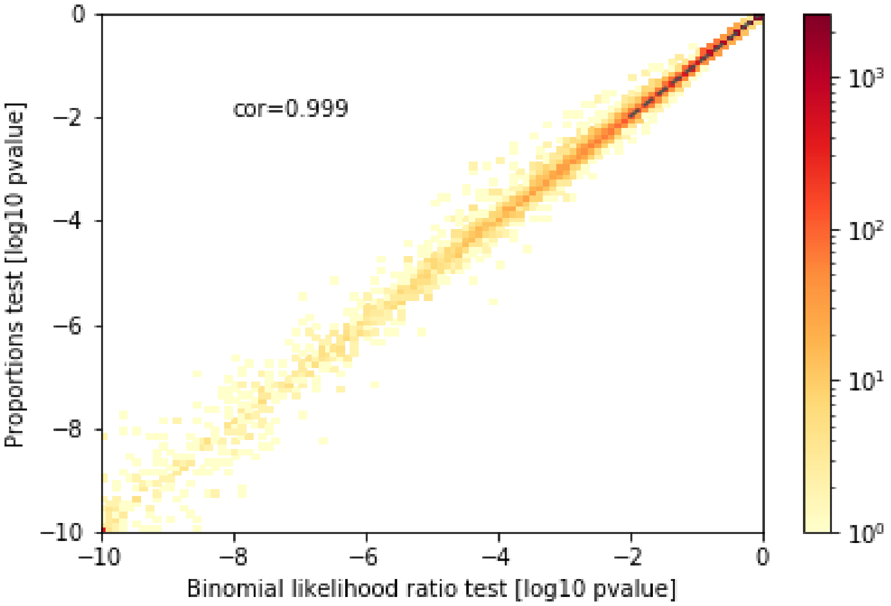

Figure S13: Comparison of binomial likelihood ratio test and two proportions z-test, for VPA vs. CTL samples.
